# A novel monoclonal antibody KMP1 has potential antitumor activity of bladder cancer by blocking CD44 in vivo and in vitro

**DOI:** 10.1002/cam4.1446

**Published:** 2018-03-25

**Authors:** Yujin Chen, Haifeng Wang, Yigang Zuo, Ning Li, Mingxia Ding, Chong Li

**Affiliations:** ^1^ Department of Urology The Second Affiliated Hospital of Kunming Medical University, Yunnan Institute of Urology Kunming 650101 China; ^2^ Kidney Center Yunnan Boya Hospital Kunming 650228 China; ^3^ Core Facility for Protein Research Institute of Biophysics Chinese Academy of Sciences Beijing 100190 China; ^4^ Beijing Jianlan Institute of Medicine Beijing 100190 China

**Keywords:** Bladder cancer, CD44, KMP1, monoclonal antibody

## Abstract

Bladder cancer becomes a serious medical and social concern due to its high recurrence and mortality rates. Thus, it is urgent to search a novel prognostic biomarker and targeted therapy with high sensitivity and specificity. In this study, we used the human bladder cancer cell line EJ as an immunogen to generate a novel mouse monoclonal antibody KMP1 that specifically bound to bladder cancer, and then, the antitumor effect of KMP1 against bladder cancer was investigated both in vivo and in vitro. The results showed that expression of the KMP1 epitope is consistent with clinical severity and prognosis of bladder cancer. Furthermore, KMP1 not only significantly inhibited the proliferation, migration, and adhesion of EJ cells in vivo, but also suppressed the xenograft tumor growth in nude mice compared with the control group treated with mIgG. Subsequently, the underlying mechanism of KMP1 against bladder cancer was explored via antigen affinity chromatography and mass spectrometry. CD44 located on the cytomembrane was found as the antigen of KMP1. Using RNA interference technology to knock down CD44 expression, we further identified that KMP1 has the antitumor activity by binding to CD44 and blocking its functions. In conclusion, KMP1 might be valuable for development as a promising specific diagnostic biomarker or targeted agent for bladder cancer.

## Introduction

Bladder cancer is one of the most common urogenital malignant tumors with frequent recurrence and poor prognosis, which make it become one of the main causes of death in the world. The incidence and death rates of bladder cancer are rising year by year. It was estimated there were 429,800 new cases and 165,100 deaths worldwide in 2012 1 and 79,030 new cases and 16,870 deaths in the United States in 2017 2. According to Globocan 2012 and the National Central Cancer Registry of China (NCCR) 2015 annual report, the overall incidence of bladder cancer was 7.68/10^5^ and the population mortality rate was 1.99/10^5^ in 2011, which makes it the 12th most common cancer in both sexes in China 3.

About 70% of patients with bladder cancer are non–muscle‐invasive bladder cancer (NMIBC) with a tendency of recurrence and 30% are muscle‐invasive bladder cancer (MIBC) associated with a high risk of death from distant metastases 4. Usually, patients with NMIBC were treated with tumor removal via the transurethral approach followed by a postoperative immediate intravesical instillation of chemotherapy or immunotherapy, while patients with MIBC were treated with radical cystectomy and perioperative chemotherapy 5. However, 31–78% of NMIBC cases recur and 17–45% of cases progress to MIBC within 5 years 6, and the therapies are only effective for 30–40% of patients with MIBC 7. These situations necessitate the development of innovative treatment strategies that can improve the outcomes of patients with bladder cancer.

To date, many immunotherapies including monoclonal antibodies, cancer vaccines, and cytokine therapies have been increasingly used for treatment of many solid tumors 8. Several monoclonal antibodies have been used in cancer treatment or tested in both preclinical and clinical trials 9, 10. However, there are few well‐validated monoclonal antibodies to improve the prognosis of bladder cancer. Recently, monoclonal antibodies blocking PD‐1 and its ligand have been demonstrated as the therapeutic strategy with spectacular effect on objective response and with sustainable clinical benefit for metastatic bladder cancer 11, 12, 13. The anti‐PD‐1 monoclonal antibody MPDL3820A was considered as an antibody drug only effective for metastatic or advanced bladder cancer in the past 30 years, but the effective ratio of clinical treatment is only 30% 11. Therefore, it is urgent to search a novel prognostic biomarker and targeted therapy with high sensitivity and specificity for bladder cancer.

In this study, we generated a novel mouse monoclonal antibody KMP1 and investigated its therapeutic efficacy against bladder cancer both in vitro and in vivo. We also explored the underlying mechanism of KMP1 against bladder cancer.

## Materials and Methods

### Cells, tissues, animals, and reagents

Human bladder cancer cell lines (EJ, BIU‐87, and T24), human cervical cancer cell line HeLa, human liver cancer cell line HepG2, and normal human bladder cell line HCV29 were all bought from American Type Culture Collection (Manassas, VA). Human bladder cancer and normal bladder tissues, human normal peripheral blood red blood cells (RBCs), and lymphocytes (LYMs) were obtained from the Second Affiliated Hospital of Kunming Medical University (Kunming, China) with informed consents, according to the approved protocol of the local committee of ethics. Cells were cultured in Roswell Park Memorial Institute (RPMI)‐1640 (Invitrogen, Carlsbad, CA, USA), supplemented with 10% fetal bovine serum (FBS) and antibiotics (GIBCO, Rockville, MD, USA) at 37°C in a humidified atmosphere of 5% CO_2_.

BALB/c normal mice and nude mice were purchased from Beijing Vital River Laboratory Animal Technology Company (China). All mice were maintained under specific pathogen‐free conditions in the Experimental Animal Center of Kunming Medical University. The animal experiments were approved by the Animal Care Committee of Kunming Medical University in accordance with Institutional Animal Care and Use Committee guidelines (IACUC).

Horseradish peroxidase (HRP)‐labeled goat anti‐mouse IgG (H+L) secondary antibody (Pierce, Rockford, IL, USA) was used for enzyme‐linked immunosorbent assay (ELISA), with murine IgG (Sigma, Munich, Germany) used as a control mIgG. Rabbit anti‐mouse IgG‐fluorescein isothiocyanate (FITC) secondary antibody (Sigma) and biotin‐conjugated goat anti‐mouse IgG (H+L) secondary antibody (Thermo Scientific, Rochester, NY, USA) were used in immunohistochemistry (IHC). Other primary reagents included a DAB HRP color development kit (Invitrogen), a mouse mAb isotyping ELISA kit (R&D, Minneapolis, MN, USA), protein A‐Sepharose (BioVision, Milpitas, CA, USA), *β*‐actin antibody (Sigma), pSuper‐puro plasmid (Merck, Billerica, MA, USA), Lipofectamine 2000 reagent (Invitrogen), avidin‐HRP antibody (Pierce), and enhanced chemiluminescence (ECL) reagents (GE Healthcare, Pittsburgh, PA, USA).

### Production, purification, and isotype of monoclonal antibody

Monoclonal antibody was produced using the hybridoma technology 14. Briefly, four normal female BALB/c mice (6–8 weeks old) were intraperitoneally injected with 1 mL of 1 × 10^7^/mL EJ cell suspension as a primary immunization, and then, the similar booster injections were performed at 3, 5, and 7 weeks after primary immunization. Three days after the fourth booster vaccination, the immunized mice were sacrificed for splenocyte collection. After fusion with 2 × 10^8^ splenocytes and 5 × 10^7^ SP2/0‐Ag14 mouse myeloma cells, the cells were washed twice with RPMI 1640. Then, 50% polyethylene glycol (PEG) 1500 (preheated to 37°C; Sigma, St Louis, MO, USA) was added to the cell pellet slowly under continuous shaking. Fused cells were selected by a hypoxanthine aminopterin thymidine (HAT) medium (Sigma), washed with RPMI 1640, and then distributed in 96‐well plates. Hybridoma cell culture supernatants were added to the cell pellet fixed with EJ cells, and then, HRP‐labeled goat anti‐mouse IgG (H+L) secondary antibody was used to screen out the positive colonies that could secrete a single antibody by ELISA. Next, female BALB/c normal mice (6–8 weeks old) were intraperitoneally injected with 1 mL of pristine (Sigma), followed by 5 × 10^6^ screened hybridoma cell suspensions. After 10 to 14 days, the mice were sacrificed, and all the ascitic fluids were harvested. Antibodies were purified from the ascitic fluids by affinity chromatography using protein A‐Sepharose and determined by sodium dodecyl sulfate–polyacrylamide gel electrophoresis (SDS–PAGE) according to the manufacturer's protocol. Afterward, the mouse monoclonal antibody isotyping ELISA kit was used to identify the purified antibody isotype.

### Flow cytometry

EJ, BIU‐87, T24, HeLa, HepG2, HCV29 cells, peripheral RBCs, and LYMs were used to determine the reactivity of KMP1 by flow cytometry. About 2 × 10^7^ EJ, BIU‐87, T24, HeLa, HepG2 cells and RBCs, and 1 × 10^5^ normal LYMs were suspended. Cells were then incubated with KMP1 (0.1 mg/mL) for 40 min on ice in phosphate buffer solution (PBS), using the murine IgG (mIgG, 0.1 mg/mL) as a negative control. After centrifugation at 400 × g for 5 min, each supernatant was discarded and the cells were washed thrice with PBS‐0.1% bovine serum albumin (BSA). After addition of an FITC‐conjugated rabbit anti‐mouse secondary antibody (100 *μ*L), the cells were incubated at 4°C in the dark for additional 30 min, washed thrice with PBS, suspended in PBS, and then measured on a BD FACSCalibur Flow Cytometer ( BD Biosciences, San Jose, CA, USA). The data were analyzed on Flow Jo 7.6.1 (Tree Star, San Carlos, CA).

### IHC sta ining

EJ, HCV29, human bladder cancer tissues, and normal human bladder tissues were used to assay the antigen‐binding site and activity of KMP1 by IHC staining. Briefly, as for IHC staining of EJ and HCV29, the cells were suspended at a density of 5 × 10^4^ in PBS and air‐dried on glass slides at room temperature for 1 h. Next, the cells were incubated with RPMI‐1640 supplemented with 20% FBS at 37°C for 24 h. After fixation in 5% paraformaldehyde for 15 min and washing with PBS, the cells on glass slides were incubated with KMP1 (1 ×10^−4^ mol/L) at 37°C for 1 h. Meantime, for IHC staining of bladder tissues, the sections of human bladder cancer tissues or normal human bladder tissues were deparaffinized, rehydrated, incubated with 3% H_2_O_2_ for 30 min, washed, and blocked with 5% goat serum (Millipore, Billerica, MA, USA) for 30 min at room temperature. After that, the sections were incubated with KMP1 (1:400) overnight at 4°C. All slides of cells and tissues were incubated with biotin‐conjugated goat anti‐mouse IgG (H+L) secondary antibody (1:50) at 37°C for 30 min and then washed thrice with PBS. The DAB HRP color development kit was used to produce a brown precipitate according to the manufacturer's instructions. The slides were examined under a light microscope.

### Soft agar colony formation assay

To investigate the impact of KMP1 on the proliferation of bladder cancer cells, we measured cloning efficiency by counting the colonies growing in soft agar. Briefly, EJ cells were seeded in 12‐well plates (300/well) and each well was treated with 0.03 mg (0.01 mg/mL) of KMP1, and the murine IgG was used as a negative control. Ten days later, the numbers of colonies was counted under the microscope.

### Wound‐healing assay

Wound‐healing assays were performed to investigate the effect of KMPI on the migration of EJ cells. EJ cells were seeded in 6‐well plates (5 × 10^4^/well) and incubated with RPMI‐1640 plus 20% FBS at 37°C for 7 days. The cells, when spreading to more than 80% of the bottom of a well, were wounded by a 200‐*μ*L pipet tip. Plates were washed with PBS to remove detached cells and then incubated with RPMI‐1640 without FBS. The cells were treated with 0.5 mg (1 mg/mL) of KMP1 or mIgG. At 0 (immediately), 6, 12, 18, and 24 h after scratching, images of the wounds were taken respectively to assess the ability of the cells to migrate into the wound area, and the migration rates were calculated.

### Cell adhesion assay

Cell adhesion assay was used to assess the effect of KMPI on the attachment of EJ cells. Briefly, 96‐well plates were incubated with Matrigel (BD Biosciences) and blocked with 10% BSA (Sigma) for 1 h. EJ cells in the logarithmic growth phase were resuspended to a density of 5 × 10^5^ cells/mL in RPMI‐1640 with 1% FBS after digestion with 1% trypsin, followed by the incubation at 37°C for 15 min. The cells were treated with 0.5 mg (1 mg/mL) of KMP1 or the control mIgG. After the unattached cells were washed away, the attached cells of each well were fixed, washed, and stained with 50 *μ*L of 0.1% (w/v) crystal violet (Sigma) for 30 min. After the uncombined dye was washed away with PBS, the stained crystal violet was destained and dissolved. ELISA microplate reader was used to measure the absorbance at 575 nm of each well. After subtracting background absorbance, results were calculated as the mean cellular adhesion rate.

### Xenograft assays in nude mice

Xenograft assays in nude mice were performed to explore the effects of KMP1 on cancerous growth in vivo. To exactly monitor the tumor growth, we transfected EJ cells with green fluorescent protein (GFP, Promega, Madison, WI, USA) for continuous visualization of tumor growth 15. EJ‐GFP cells in the logarithmic growth phase were resuspended in D‐Hank's buffer (Invitrogen). Then, twenty female 6‐week‐old BALB/c nude mice with a mean body weight of 16 g were inoculated subcutaneously with 1.5 × 10^6^ EJ‐GFP cells in the right dorsal flank. Three days later, the mice were randomly divided into KMP1 group and control mIgG group (each 10 mice) and were intraperitoneally injected with 200 ul (1 mg/mL) of KMP1 or mIgG twice a week for 5 weeks. Tumor sizes were measured using a caliper at a 2‐day interval for 40 days after inoculation, and body weights were monitored once a week. Tumor volume (*V*) was calculated as *V* = *π*/6 × (larger diameter) × (smaller diameter)^2^ 16. At the first and 5th week after inoculation, the tumorigenesis and metastasis of tumor were accurately monitored by LB983 noninvasive small animal living fluorescence imaging system (Berthold Technologies GmbH & Co KG, Germany). All mice were sacrificed 40 days after inoculation, and then, the tumors were resected, weighed, and formalin‐fixed for histology and IHC. Tumor inhibition rate (TIR) was determined as TIR (%) = [1 − (mean tumor weight of KMP1 group/ mean tumor weight of control group)] × 100%.

### Histology and IHC of Xenograft

Formalin‐fixed tissues were paraffin‐embedded, sectioned serially (4 *μ*m), and mounted on slides. The slides were deparaffinized and stained with hematoxylin/eosin (H&E) for histology. The adjacent slides were stained with KMP1 for IHC as described above.

### Antigen purification, identification, and verification

The epitope recognized by KMP1 was investigated with protein A‐Sepharose and mass spectrometry (MS). Briefly, 5 × 10^7^ EJ cells in the logarithmic growth phase were homogenized in 0.5 mL of an ice‐cold cell lysis buffer, incubated for 30 min on ice, and then centrifuged at 16,100 × g for 10 min at 4°C. The supernatants were removed to a protein A‐Sepharose column with 0.1 mg of KMP1 (1 mg/mL). The bound proteins were eluted with 0.2 M glycine at pH 2.8 and then neutralized with 1 M Tris–HCl buffer at pH 8.0. The eluates were separated and concentrated by gel filtration and resolved by 10% SDS–PAGE under reducing conditions. The gels were fixed and stained with silver, and then, distinct proteins bands were obtained for MS.

After that, to verify the KMP1‐recognized epitope, we performed RNA interference technology to silence CD44 in EJ cells and detected the antibody‐binding activity of KMP1 using Western blotting, flow cytometry, and ELISA. The RNA sequence against CD44 for RNAi was designed on basis of pSuper system instructions (Oligoengine, Seattle, WA, USA) and cloned into a pSuper‐puro vector. The short hairpin RNA (shRNA) pSuper‐puro‐CD44 (pSuper‐puro‐CD44‐shRNA) that expressed 19‐nt hairpin‐type with a 9‐nt loop was constructed. The sequence encoding CD44 shRNA was as follows: 5′‐GAAGGGCACGTGGTGATTC‐3′ (sense); 5′‐TCCGACAACATGTAAAGGA‐3′ (antisense); and 5′‐TTCAAGAGA‐3′ (loop). EJ cells were transfected with pSuper‐puro‐CD44‐shRNA (shCD44‐EJ) using Lipofectamine 2000 as described by the manufacturer's instruction, with the empty pSuper‐puro vector (shCtrl‐EJ) used as a control. The efficiency of CD44 silencing after transfection was examined on a 7900HT quantitative real‐time polymerase chain reaction (qRT–PCR) System (Applied Biosystems, Foster City, CA, USA). *β*‐Actin (Sigma) was used as the internal control. The forward and reverse primers sequences were as follows: 5′‐GCAGTCAACAGTCGAAGAAGG‐3′, 5′‐TGTCCTCCACAGCTCCATT‐3′ (CD44); 5′‐GAAGGTGAAGGTCGGAGTC‐3′, 5′‐GAAGATGGTGATGGGATTTC‐3′ (*β*‐actin). All experiments were repeated at least three times. The relative expression of CD44 mRNA was calculated with the 2^−△△CT^ method 17.

Western blotting was performed to examine the protein expression of CD44 recognized by KMP1 in the CD44‐silenced EJ cells (shCD44‐EJ). For this purpose, proteins in shCD44‐EJ cells and shCtrl‐EJ cells were separated by SDS–PAGE, transferred onto nitrocellulose membranes (Millipore), and incubated with primary antibody (20 µg KMP1) at 4°C for 2 h and avidin‐HRP antibody as the secondary antibody (Pierce) at 37°C for 30 min. Then, ECL reagents were added. *β*‐Actin was probed as a control for normalization. Relative quantitative analyses were performed by ImageJ (National Institutes of Health, Bethesda, Maryland, USA).

Finally, shCD44‐EJ cells were managed according to the previous procedure for flow cytometry and seeded in 96‐well plates (500/well) for ELISA. ShCtrl‐EJ cells were used as a control, and mIgG was used as a negative control. For ELISA, EJ cells were incubated in RPMI‐1640 with 20% FBS, treated with 0.5 mg of 1 mg/mL KMP1, fixed with 4% paraformaldehyde, washed with PBS, and administered with 200 *μ*L HRP‐labeled goat anti‐mouse IgG(H+L) secondary antibody at 37°C for 30 min. After that, the absorbance at 490 nm of each well was measured.

### Statistical analysis

Data were presented as mean ± SD. Statistical analysis was performed using the Student's *t* test. Values with *P *<* *0.05 were considered statistically significant. SPSS v21.0 software (IBM, Armonk, NY, USA) was used for all data analysis.

## Results

### Generation and subtype of KMP1

**Figure 1:**
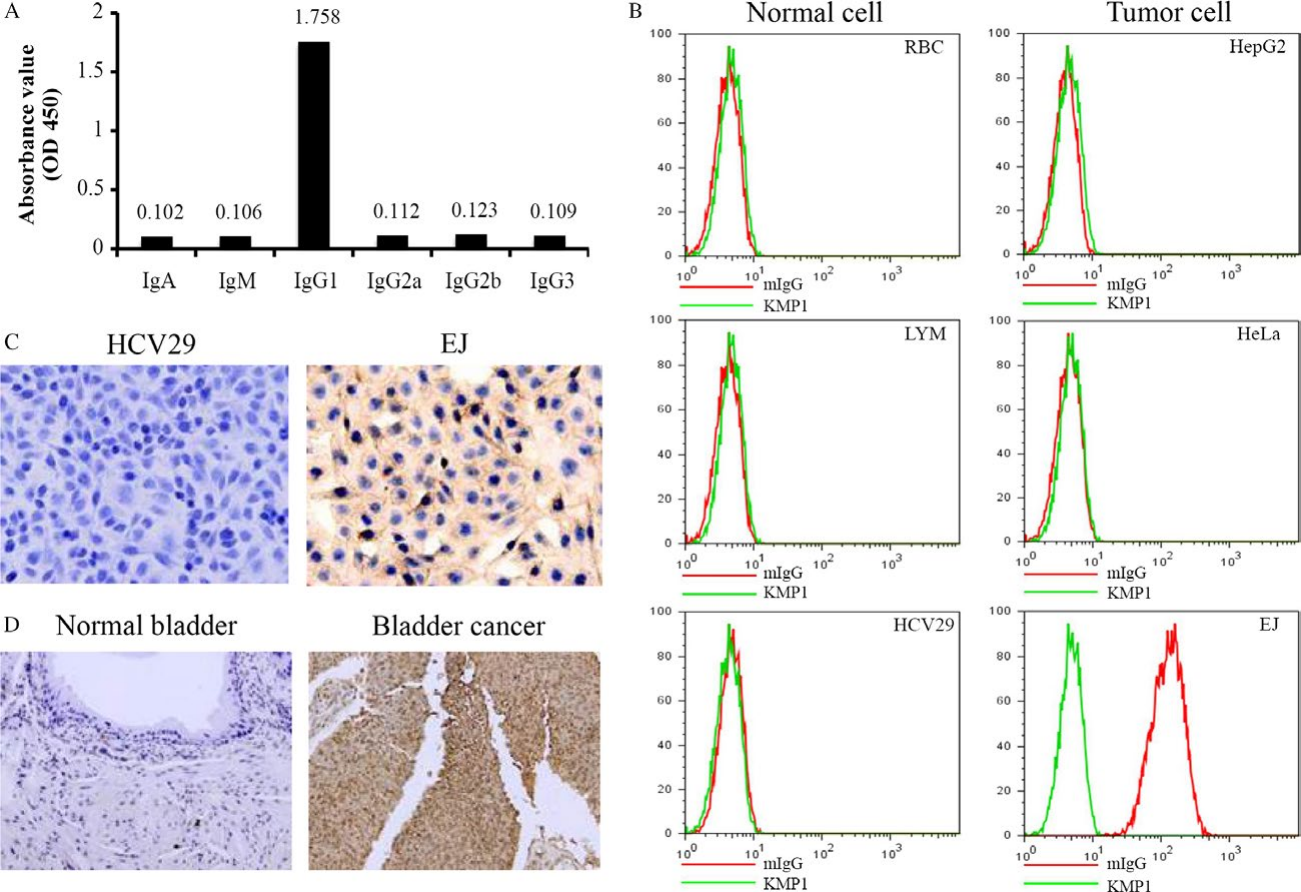
Subtype and tumor specificity of KMP1 for bladder cancer. (A) The isotype of obtained monoclonal antibody KMP1 was determined as IgG1 by ELISA. (B) KMP1 could specifically bind to bladder cancer cell lines EJ, BIU‐87, and T24, but could not react with human cervical cancer cell line HeLa, human liver cancer cell line HepG2, normal human bladder cell line HCV29, peripheral RBC, or LYM by flow cytometry. (C) KMP1 recognized its antigen on the bladder cancer EJ cells, but not on the normal bladder HCV29 cells by immunohistochemistry assays. The KMP1‐positive areas expressed as deep brown were mainly located on the cytomembrane. (Magnification, ×400). (D) KMP1 recognized its antigen on the human bladder cancer tissue, but not on the human normal bladder tissue. (Magnification, ×200).

The EJ cells were used as immunogen to obtain hybridoma cells producing mouse monoclonal antibodies. After positive hybridoma colonies that could secrete a single antibody were screened out, the high‐affinity monoclonal antibody KMP1 was selected and purified by protein A‐Sepharose affinity chromatography. The isotype of KMP1 was determined as IgG1 by ELISA (Fig. 1A).

### Binding specificity of KMP1 to bladder cancer

We examined the activity of KMP1 binding to tumor cells and normal cells by flow cytometry in vitro. Results showed that KMP1 could bind to the human bladder cancer cells such as EJ, BIU‐87, and T24, but could not react with other tumor cells (Hela, HepG2) or normal cells (HCV29, RBCs and LYMs) (Fig. 1B, Data of BIU‐87 and T24 are not shown). In order to further investigate the specificity of KMP1 binding to bladder cancer cells and tissues, we performed IHC staining with KMP1. Normal bladder cells HCV29 and bladder tissues were used as the controls. KMP1 positively stained both EJ cells and human bladder cancer tissues, but not HCV29 cells or human bladder tissues (Fig. 1C,D). In addition, the KMP1‐positive areas stained as deep brown were mainly distributed on the cytomembrane (Fig. 1C). These results indicate that KMP1 could specifically recognize antigen located on the cytomembrane in bladder cancer.

### Expression of the KMP1 epitope is consistent with clinical severity and prognosis of bladder cancer

**Figure 2:**
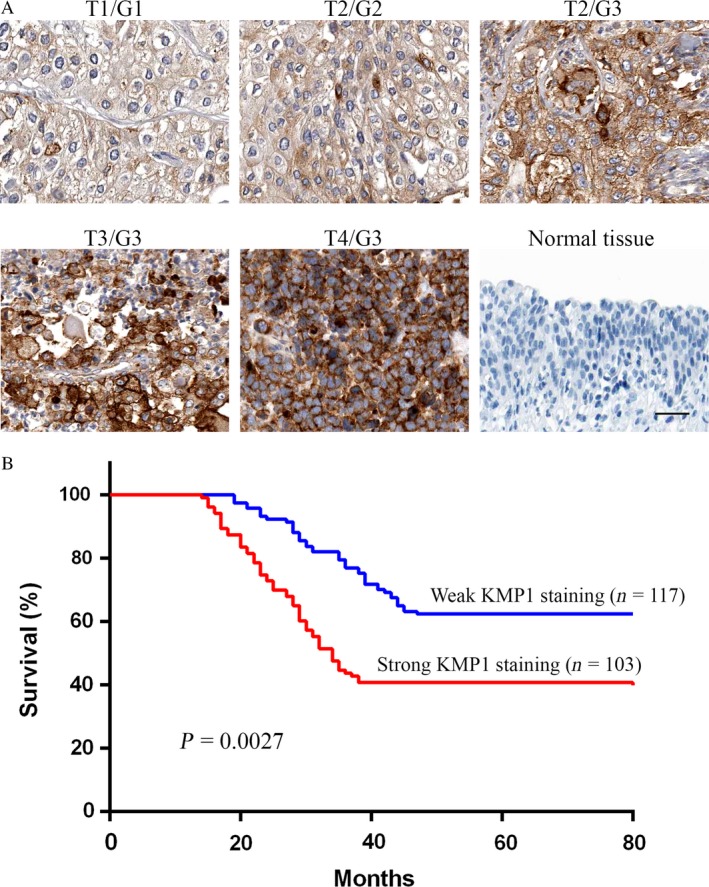
Expression of the KMP1 epitope is consistent with clinical severity and prognosis of patients with bladder cancer. (A) The expression level of the KMP1 epitope is in agreement with clinical severity. Serial sections of bladder cancer biopsies were stained with KMP1 by immunohistochemistry analysis. Normal bladder biopsy from a healthy person was not stained with KMP1. (B) Relationship between the expressions of KMP1 epitope and prognosis in radical cystectomy patients. The patients with high expressions of the KMP1 epitope had significantly worse prognosis than those with low expressions of the KMP1 epitope (*P *=* *0.0027); *n*, patient number.

To further investigate whether the KMP1 epitope is associated with clinical severity, we examined its expression in the patients with bladder cancer through immunohistochemical staining. We detected bladder tumor tissues from biopsies with classification of Grade (G1–G3) and Stage (T1–T4) among 220 patients with bladder cancer. Low grades G1–G2 and low stages Ta–T1 were weakly stained (Fig. 2A). Severe patients with high grade (G3) and high stages (T3–T4) showed intense staining of KMP1. Normal bladder tissues were not stained as a negative control. The expression levels of KMP1 epitope correlated with pathological grades and tumor stages of bladder cancers. We analyzed the relationship between expression of the KMP1 epitope and clinical–pathological features in patients with bladder cancer. The histologically high grade, deeply invasive, and lymphatic‐invasive bladder cancers demonstrated significantly higher expression of the KMP1 epitope than the low‐grade, superficial, and nonlymphatic‐invasive cancers (Table 1). There were no significant correlations between expression of the KMP1 epitope and age, sex, and tumor number (all *P *>* *0.05) (Table 1). To further determine the association between the KMP1 epitope expression and prognosis, we completed 80‐month follow‐up of the above 220 bladder cancer patients with radical cystectomy. All 220 patients with bladder cancer were grouped according to high or low expression of the KMP1 epitope, and the mean follow‐up time was similar between the two groups (89.6 vs. 85.4 months, *P *=* *0.637) (Table 1). Intriguingly, the patients with high expression of the KMP1 epitope had significantly worse prognosis than patients with low expression (*P *=* *0.0027) (Fig. 2B).

**Table 1:** Relationship between the expression levels of KMP1 antigen and clinical–pathological features of bladder cancers

Total		Patients	Expression of aberrantly		
			KMP1 antigen		
			High	Low	*P*
Age (years)	Mean	63.9	62.6	65.2	0.537
Sex (*n*)	Male	123	57	66	0.712
	Female	97	45	52	
Tumor stage (*n*)	T1	82	27	55	<0.001
	T2	73	35	38	
	T3	53	31	22	
	T4	12	10	2	
Grade (*n*)	G1 or G2	143	47	96	<0.001
	G3	77	56	21	
Number of tumors (*n*)	Solitary	163	73	90	0.083
	Multiple	57	30	27	
Lymphatic invasion (*n*)	Negative	73	23	50	<0.005
	Positive	89	63	26	
	Unknown	58	17	41	
Follow‐up (months)	Mean	87.5	89.6	85.4	0.637

### KMP1 inhibits proliferation, migration, and adhesion of bladder cancer cells in vitro

**Figure 3:**
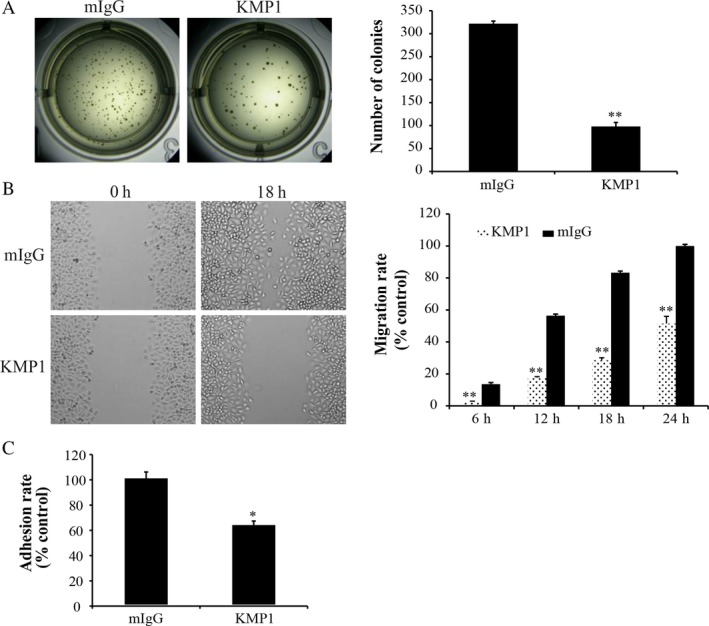
Effect of KMP1 on the proliferation, migration, and adhesion of bladder cancer cells in vitro. (A) Representative images of the soft agar colony formation assay showed that the proliferation of KMP1‐treated cells was significantly suppressed. (B) KMP1 treatment reduced the migration of EJ cells through wound‐healing experiments. (C) The adhesion rate in KMP1‐treated cells was lower than that in mIgG‐treated cells by crystal violet adhesion assay. Each bar was expressed as the mean ± standard deviation of three experiments, mIgG as a control. *P <* *0.01; **P <* *0.001, versus mIgG control.

To explore whether KMP1 plays a role as an efficacious antitumor target for bladder cancer, we examined the influence of KMP1 on EJ cells in vitro. KMP1 treatment significantly decreased colony formation of EJ cells compared with mIgG treatment (98 ± 8.8 vs. 322 ± 5.5 colonies/well, *P *<* *0.001) (Fig. 3A). Wound‐healing assay showed KMP1 treatment significantly suppressed cell migration in human bladder cancer cell line EJ. Compared with mIgG treatment, the cell migration rates were obviously lowered after treatment with KMP1 at 6, 12, 18, and 24 h (all *P *<* *0.001) (Fig. 3B). Crystal violet adhesion assay showed similar results. After KMP1 treatment, cell adhesion rate reduced to 63% compared with mIgG treatment (*P *<* *0.01) (Fig. 3C). These data indicate that KMP1 significantly inhibits proliferation, migration, and adhesion of human bladder cancer cell line EJ cells in vitro.

### KMP1 inhibits the growth of subcutaneous xenografts of bladder cancer in vivo

**Figure 4:**
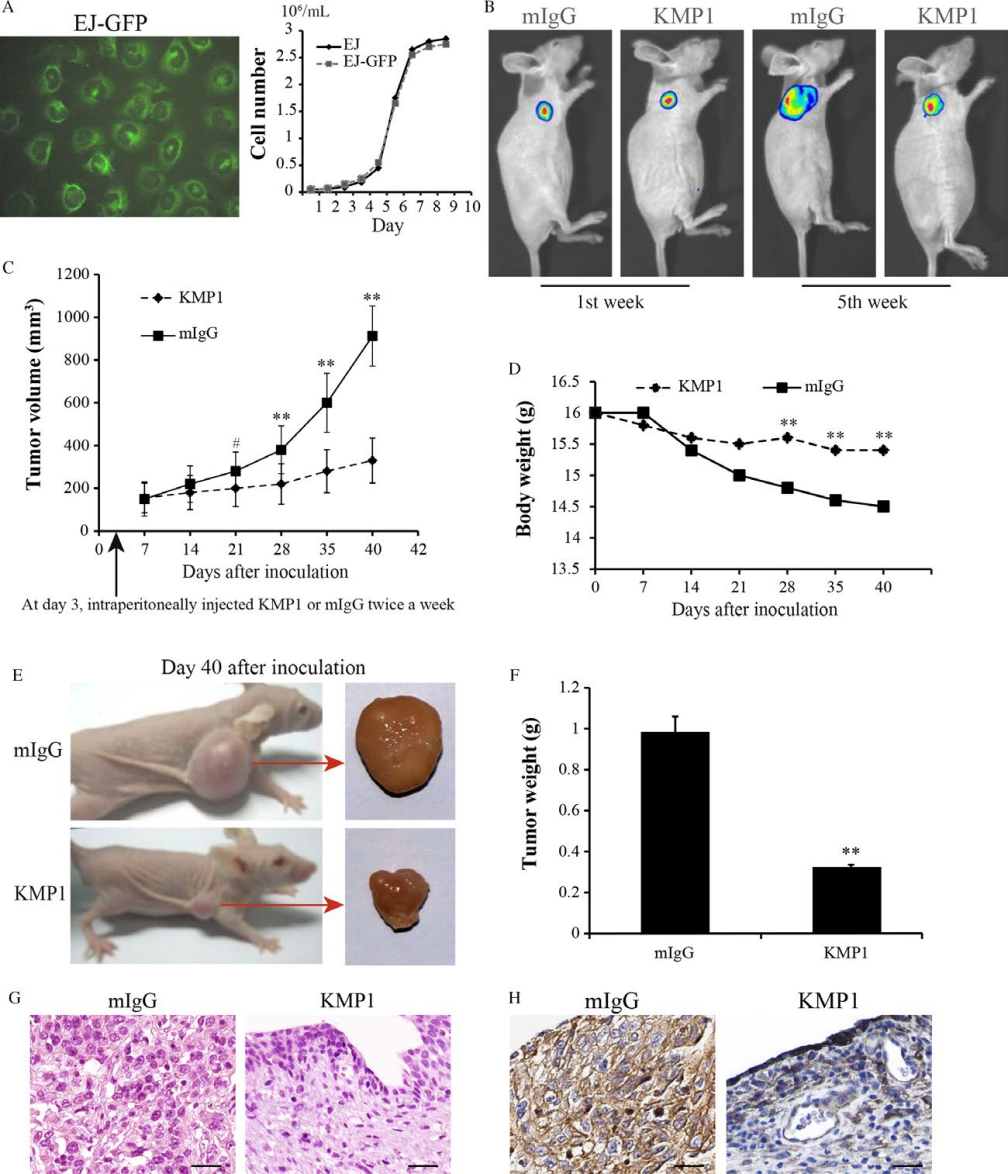
Therapeutic efficacy of KMP1 against bladder tumor growth in a xenograft mouse model. The mice xenograft models were established by the inoculation of EJ‐GFP cells in BALB/c nude mice. Three days after inoculation, KMP1 or mIgG (control) was intraperitoneally injected into the tumor‐bearing mice (10 mice/group), and tumor growth was monitored for 40 days. (A) EJ‐GFP cells presented green fluorescence under the fluorescent microscope, and there was no difference in growth curves between EJ‐GFP cells and parental EJ cells. (B) Representative bioluminescence images of tumors in mice at the first week and 5th week after inoculation. No metastasis of tumor was found in both KMP1‐treated and control mIgG‐treated mice. (C) Subcutaneous xenografts in KMP1‐treated mice grew more slowly than those in the mIgG‐treated mice, and there were significant differences since the 3rd week after the application of KMP1 or mIgG. (D) Body weights between the two groups were significant differences at day 28, 35, and 40. (E) Representative macroscopic appearance of tumor‐bearing mice and the resected tumor 40 days after inoculation. (F) The resected tumor weight in KMP1‐treated mice was obviously lower than that in control mIgG‐treated mice. (G) Representative H&E images of the resected tumors. (Magnification, ×400). (H) Representative Immunohistochemical staining imagines of the resected tumors with KMP1. The KMP1‐positive area is expressed as deep brown. (Magnification, ×400). Data are represented as mean ± standard deviation, mIgG as a control. ^#^ *P *<* *0.05; **P <* *0.001, versus mIgG control.

To evaluate the antitumor effect of KMP1 in vivo, we established EJ‐GFP cells and generated xenografted tumor models by subcutaneously injecting EJ‐GFP cells into the right dorsal flank of nude mice. As shown in Figure 4A, the growth curve of EJ‐GFP cells was similar to that of parental EJ cells. Three days after subcutaneous inoculation with EJ‐GFP cells, KMP1 or mIgG was intraperitoneally injected into the tumor‐bearing mice (10 mice/group) twice a week for 5 weeks. After transplantation, tumor sizes were measured using a caliper every three days for 40 days, and bioluminescence images of tumors were obtained at the first and 5th weeks (Fig. 4B). Since the 3rd week after treatment, the sizes of subcutaneous xenografts were significantly reduced in KMP1‐treated mice compared with mice treated with control mIgG (*P *<* *0.05 at day 21, *P *<* *0.001 at another time points after 21 days) (Fig. 4C). During the experiments, no metastasis of tumors was found in either group in terms of bioluminescence results (Fig. 4B), but there were significant differences in body weights between the two groups (*P *<* *0.001 at day 28, 35, and 40) (Fig. 4D). At the end of observation period (40 days after inoculation), the tumor inhibition rate for KMP1 was 67.11%, indicating that KMP1 treatment remarkably inhibited tumors in mice bearing EJ cells compared with mIgG treatment (*P *<* *0.001) (Fig. 4E,F). With KMP1 treatment, only few cancer cells and KMP1‐positive staining cells were detectable in the EJ cell implanted locations with immunohistochemical staining and hematoxylin and eosin (H&E) counterstaining (Fig. 4G,H, right). However, tumors were apparent in mIgG‐treated mouse bladders and large numbers of cancer cells were confirmed by histologic staining (Fig. 4G,H, left). Taken together, KMP1 significantly suppressed the growth of bladder cancer in vivo.

### KMP1 recognizes CD44 epitope located on the cell surface in bladder cancer

**Figure 5:**
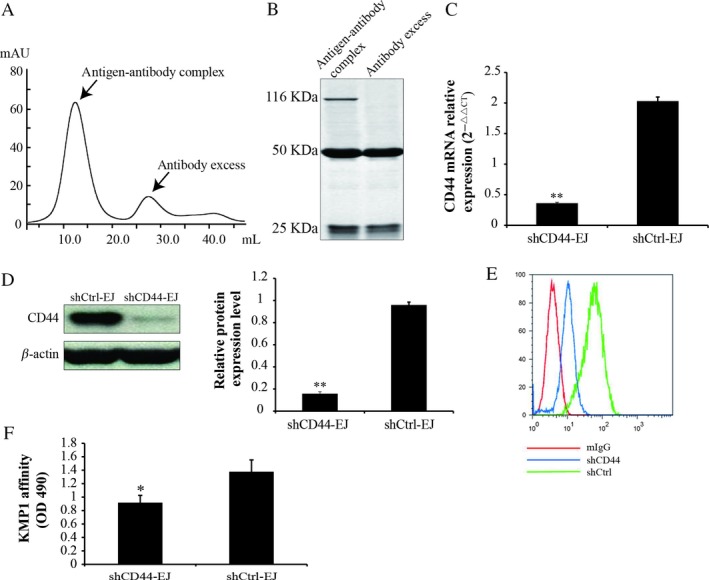
KMP1 specifically recognized CD44 epitope located on the cell surface in bladder cancer. (A) To investigate the epitope recognized by KMP1, the supernatants after EJ cell lysis were applied to protein A‐Sepharose column with KMP1. Two peaks were obtained from the eluates by Sephacryl S200. (B) Antigen–antibody complex peak and antibody excess peak obtained from the eluates were resolved by 10% SDS–PAGE and stained with silver. (C) qRT–PCR analysis of CD44 expression in EJ cells that were transfected with pSuper‐puro‐CD44‐shRNA (shCD44‐EJ) or the empty pSuper‐puro vector (shCtrl‐EJ). (D) Western blot imagines indicated that the band using KMP1 as a primary antibody was markedly thinned in the CD44‐silenced EJ cells versus the shCtrl‐EJ cells. (E) shCD44‐EJ and shCtrl‐EJ cells were analyzed by flow cytometry after staining with KMP1, and nontransfected EJ cells were stained with mIgG as a negative control. (F) ELISA analysis of KMP1 recognized CD44 levels in shCtrl or shCD44‐EJ cells. Each bar is expressed as the mean ± standard deviation of three experiments. *P < 0.01; **P < 0.001, versus shCtrl‐EJ control.

To investigate the KMP1‐recognized epitope, we applied the supernatants after EJ cell lysis to a protein A‐Sepharose column with KMP1. Antigen–antibody complex peak and antibody excess peak were obtained from the eluates by Sephacryl S200, and the two peaks were resolved by 10% SDS–PAGE and stained with silver (Fig. 5A,B). Procured antigen–antibody complex protein bands analyzed by MS were showed in Table 2. We found that the antigen recognized by KMP1 was the cell surface glycoprotein CD44. However, the molecular weight of CD44 bound to KMP1 was 116 kDa, which was inconsistent with MS‐predicted molecular mass of CD44 (85.756 kDa) (Fig. 5B and Table 2).

**Table 2:** Complete information of the detected peptides for antigen–antibody complex proteins bands by mass spectrometry

	Reference				Score	Coverage	MW	Accession	Peptide (Hits)	Count
	Scan(s)	Peptide	MH+	z	XC	DeltaCn	Sp	RSp	lons	
1	Cell surface glycoprotein CD44 [Homo sapiens]				96.30		85756.0	6006011	10 (91,000)	
	4184	R.GLCLVPLSLAQIDL.D	1431.65517	2	3.44	0.40	817.8	1	16/24	1
	4236	R.FNSTLPTMAQME.R	1569.65696	2	4.21	0.44	1330.6	1	20/24	1
	4704	R.TSQYDTYC.E	974.18150	2	3.14	0.44	1554.8	1	15/16	1
	5265	R.YPSNPTDDDVSSG*K.T	1692.93394	2	3.01	0.19	768.3	1	16/30	1
	5304	K.TFHPSGGSHTTHGS.Q	1775.94547	2	3.88	0.57	1213.9	1	21/32	1
	5550	R.ALILAVCIAVNSRRRCG.R	1802.04787	2	5.98	0.57	3828.0	1	29/32	1
	5600	R.LVINSGNGAVEDRKPS.T	1676.93902	2	4.26	0.49	1569.4	1	22/30	1
	6376	K.GEASKSQEM.D	1230.50277	2	3.48	0.26	1398.1	1	16/18	1
	6582	R.SETPDQFMTADETRNL.C	2135.41062	2	4.61	0.55	1509.1	1	21/36	1
	5265	R.QNVDMKIGV.T	1692.93394	2	2.45	0.48	581.1	2	15/30	1
2	Keratin 15 [Homo sapiens]				20.17		49198.4	24430190	2 (20,000)	
3	Keratin 6B [Homo sapiens]				20.16		59999.5	5031841	2 (20,000)	
4	Keratin 1B [Homo sapiens]				20.15		61801.9	45597458	2 (20,000)	
5	Cardiac muscle alpha actin 1 proprotein [Homo sapiens]				18.20		42019.8	4885049	2 (11,000)	
	6896	K.LCYVALDFENEM*ATAASSSSLEK.S	2553.77029	3	3.92	0.55	1350.6	2	31/88	3
	5424	K.SYELPDGQVITIGNER.F	1791.93998	2	2.37	0.28	646.0	1	17/30	8
6	senne (or cysteine) proteinase inhibitor, clade B (ovalbumin), Member				18.18		44854.7	28076869	2 (11,000)	
	4784	K.FYQTSVESTDFANAPEESR.K	2179.24215	2	2.46	0.63	892.5	2	17/36	
	7442	K.FMFDLFQQFR.K	1379.61046	2	3.61	0.46	1331.8	1	15/18	1

To further verify CD44 as the antigen epitope recognized by KMP1, we silenced CD44 expression in EJ cells via pSuper‐puro‐CD44‐shRNA transfection, and the empty pSuper‐puro vector (shCtrl‐EJ) as a control. CD44 expression in shCD44‐EJ cells was markedly knocked down by 82.67% (*P *<* *0.001) (Fig. 5C). The CD44 silenced EJ cells (shCD44‐EJ) and shCtrl‐EJ cells were administrated with KMP1 antibody followed by Western blot, flow cytometry, and ELISA. Western blot and flow cytometry analyses showed that CD44 knockdown extremely reduced the KMP1 staining signal of EJ cells compared with the shCtrl‐EJ cells, using mIgG as a negative control (*P *<* *0.001) (Fig. 5D,E). Similar results were obtained by ELISA (*P *<* *0.01) (Fig. 5F). These results verify that the epitope specifically recognized by KMP1 was the cell surface glycoprotein CD44.

## Discussion

Bladder cancer remains one major health problem worldwide due to its high incidence, recurrence, and mortality rates, as well as a long‐term insidious tumor progression. Nowadays, standard bladder cancer treatment guidelines include tumor resection complemented by cisplatin‐based chemotherapy or immunotherapy. However, cisplatin‐based chemotherapy is only effective in 30–40% of patients with MIBC 7, and the 1‐year recurrence‐free rate of patients with NMIBC remains 21–48% after monotherapy or multidrug combination chemotherapy, including cisplatin, gemcitabine, and mitomycin C 18, 19, 20. Bacillus Calmette–Guerin (BCG), the most common immune‐based therapy for NMIBC, has a 2‐year failure rate of 37–45% 21. Therefore, immunotherapeutic approaches for bladder cancer, particularly therapies based on monoclonal antibody, have gained increasing attention due to their specificity and efficiency 9, 10, 11. Considering the importance of monoclonal antibodies for management of bladder cancer, we aimed at finding a new monoclonal antibody with high specificity and efficiency to improve the prognosis of bladder cancer.

In this study, we successfully developed a novel murine IgG1 monoclonal antibody KMP1 using the highly invasive human bladder cancer cell line EJ as an immunogen. The murine mIgG was used as a control for further experiments. We found that KMP1 was specifically reactive with bladder cancer cells (EJ, BIU, and T24) and tissues, but unreactive with other tumor cells, normal cells, or bladder tissues. The higher expression levels of KMP1 epitope were significantly correlated with histologically high grade, poor tumor stages, deeply invasive and lymphatic‐invasive, and worse prognosis of bladder cancers. Moreover, KMP1 significantly inhibited bladder cancer both in vitro and in vivo. Affinity chromatography and MS showed that KMP1 specifically bound to the cell surface glycoprotein CD44. Then, the ability of KMP1‐specific binding to CD44 was verified using RNAi technology to knock down the CD44 expression in EJ cells.

CD44 is a multifunctional cell surface glycoprotein belonging to the cell adhesion molecule family and plays a vital role in a variety of cellular functions, including regulation of cell adhesion, proliferation, migration, angiogenesis, differentiation, and matrix cell signaling processes in collaboration with other cellular proteins 22. Hundreds of CD44 isoforms with a molecular mass from 80 to 250 kDa can be generated by alternative splicing and posttranslational modifications of CD44. These posttranslational modifications in addition to CD44s cover N‐linked or O‐linked glycosylation, glycosaminoglycan attachments, and chondroitin sulfate side‐chain connection 23, which can more than double the molecular weight of the protein 24. To date, dozens of CD44 isoforms have been discovered, including CD44v1–v12, with the most prolific isoform being the 85‐ to 95‐kDa glycoprotein known as CD44 standard (CD44s) 22, 25, 26. Numerous studies showed that CD44 and its isoforms, such as CD44s, CD44v6, and CD44v9, were upregulated and correlated with recurrence and metastasis in various cancers, including hepatocellular carcinoma 27, gastric and colon cancer 28, 29, breast cancer 30, 31, 32, urothelial carcinoma 33, 34, 35, 36, and so on. However, it has not been shown that a specific CD44 variant isoform is associated with a certain tumor type. Hence, CD44s or CD44v are not specific enough even or unique for a particular type of cancer.

Recent studies showed that aberrant glycosylation has been involved in tumorigenesis of several tumors, and a unique tumor‐specific glycopeptide antigen could be recognized by its corresponding antibody 37, 38. Consistent with these studies, CD44 glycosylation in different cells is significantly distinct. Glycosylation patterns of CD44 isoforms diversify mainly depending on cell type and the growth conditions involved 39. For example, variant isoforms of CD44 are P‐selectin and l‐selectin ligands on colon carcinoma cells, possibly owing to O‐linked glycosylation 40. Alves et al. found that LS174T CD44 binding to fibrin is dependent on O‐glycosylation of CD44, whereas binding of CD44s from HL‐60 cells to both immobilized fibrin and fibrinogen absolutely required N‐linked glycans 41. Tang et al. 42 demonstrated that the interaction between CD44s and LSECtin is dependent on the N‐glycosylation of CD44s.

Besides an adhesion molecule, CD44 also serves as a coreceptor for other signaling receptors, such as tyrosine kinase mesenchyme–epithelial transition factor receptor, epidermal growth factor receptor, and tumor growth factor‐*β* 43. In addition, CD44 ligation by anti‐CD44 mAb can inhibit tumor progression and induce differentiation or apoptosis of tumor cells, such as leukemic cells, cancer stem cells^44^, ^45^, and lung cancer cells 46. These evidences highlight the importance of CD44 as a potential anticancer therapeutic target 47.

Here, we generated a new mAb KMP1, which specifically recognized CD44 epitope on bladder cancer cells, and the antitumor effects of KMP1 were very clear at three weeks after EJ cells inoculation. These results suggested that KMP1 can be regarded as a promising specific anti‐CD44 monoclonal antibody for bladder cancer. However, we also observed that the molecular weight of CD44 bound to KMP1 was larger than its theoretically predicted molecular weight (116 kDa as estimated by SDS–PAGE and 85.756 kDa as predicted by MS). In a recent published review, Martin et al. introduced that the degree of glycosylation can give rise to CD44 molecules with a molecular mass ranging from 80 to 250 kDa, due to extensive posttranslational modification, resulting in the attachment of numerous carbohydrates to N‐ and O‐linked glycosylation sites of the extracellular domain 48. Our previous studies showed that glycopeptide‐preferring polypeptide GalNAc transferase 1 (ppGalNAc T1) mRNA was highly expressed in bladder tumor tissues and bladder cancer stem cells, and GALNT1‐positive staining in bladder cancer tissues was related to CD44 staining 49. Therefore, we propose that CD44 specifically recognized by KMP1 might undergo O‐linked glycosylation mediated by ppGalNAc T1 in bladder cancer.

We previously developed monoclonal antibody BCMab1 against aberrantly glycosylated integrin *α*3*β*1 to play antitumor role in bladder cancer in vivo and in vitro, with another bladder cancer cell line T24 as an immunogen 50. In parallel to CD44, the integrin family is another group of cell adhesion molecules and consists of noncovalently connected *α* and *β* chains, including integrins *α*3*β*1, *α*6*β*1, *α*7*β*1, *α*2*β*1, and *α*5*β*1 51. However, integrins predominantly recognize extracellular protein scaffolds such as interstitial collagen 51, while CD44 preferentially binds to extracellular carbohydrate polymers such as glycoproteins, glycosaminoglycans, and hyaluronic acid 22. Besides, our previous research had already shown that EJ cells had higher reproductive and invasion activities than T24 cells 52. Therefore, we propose that the two monoclonal antibodies recognized different specific antigenic epitopes of adhesion molecules in bladder cancer cells, due to using the human bladder cancer cell lines with different invasive abilities as immunogens.

In conclusion, we generated a novel monoclonal antibody KMP1, which specifically recognized CD44 epitope on bladder cancer cells and had specific antitumor potential both in vivo and in vitro. The highly specific binding of KMP1 to CD44 may be due to O‐linked glycosylation of CD44 mediated by ppGalNAc T1, suggesting that KMP1 will probably be a potential specific therapeutic activity and diagnostic biomarker or targeted agent for bladder cancer. Further studies are needed to explore the exact glycosylation mechanisms of CD44 bound to KMP1, and to develop humanized KMP1 antibody as well as KMP1–drug conjugate for rational and safe antibody therapeutics.

## Conflict of Interest

The authors declare no competing interests.

## References

[1] Torre, L. A. , F. Bray , R. L. Siegel , J. Ferlay , J. Lortet‐Tieulent , and A. Jemal . 2015 Global cancer statistics, 2012. CA Cancer J. Clin. 65:87–108. 25651787 10.3322/caac.21262

[2] Siegel, R. L. , K. D. Miller , and A. Jemal . 2017 Cancer statistics, 2017. CA Cancer J. Clin. 67:7–30. 28055103 10.3322/caac.21387

[3] Ferlay, J. , I. Soerjomataram , R. Dikshit , S. Eser , C. Mathers , M. Rebelo , et al. 2015 Cancer incidence and mortality worldwide: sources, methods and major patterns in GLOBOCAN 2012. Int. J. Cancer 136:E359–E386. 25220842 10.1002/ijc.29210

[4] Kaufman, D. S. , W. U. Shipley , and A. S. Feldman . 2009 Bladder cancer. Lancet 374:239–249. 19520422 10.1016/S0140-6736(09)60491-8

[5] Smith, A. B. , A. M. Deal , M. E. Woods , E. M. Wallen , R. S. Pruthi , R. C. Chen , et al. 2014 Muscle‐invasive bladder cancer: evaluating treatment and survival in the National Cancer Data Base. BJU Int. 114:719–726. 24325202 10.1111/bju.12601

[6] Sylvester, R. J. , van der Meijden A. P. , W. Oosterlinck , J. A. Witjes , C. Bouffioux , L. Denis , et al. 2006 Predicting recurrence and progression in individual patients with stage Ta T1 bladder cancer using EORTC risk tables: a combined analysis of 2596 patients from seven EORTC trials. Eur. Urol. 49:466–477. 16442208 10.1016/j.eururo.2005.12.031

[7] Shah, J. B. , D. J. McConkey , and C. P. Dinney . 2011 New strategies in muscle‐invasive bladder cancer: on the road to personalized medicine. Clin. Cancer Res. 17:2608–2612. 21415213 10.1158/1078-0432.CCR-10-2770

[8] Mellman, I. , G. Coukos , and G. Dranoff . 2011 Cancer immunotherapy comes of age. Nature 480:480–489. 22193102 10.1038/nature10673 PMC3967235

[9] Scott, A. M. , J. D. Wolchok , and L. J. Old . 2012 Antibody therapy of cancer. Nat. Rev. Cancer 12:278–287. 22437872 10.1038/nrc3236

[10] Adler, M. J. , and D. S. Dimitrov . 2012 Therapeutic antibodies against cancer. Hematol. Oncol. Clin. North Am. 26:447–481, vii. 22520975 10.1016/j.hoc.2012.02.013 PMC3334873

[11] Powles, T. , J. P. Eder , G. D. Fine , F. S. Braiteh , Y. Loriot , C. Cruz , et al. 2014 MPDL3280A (anti‐PD‐L1) treatment leads to clinical activity in metastatic bladder cancer. Nature 515:558–562. 25428503 10.1038/nature13904

[12] Rosenberg, J. E. , J. Hoffman‐Censits , T. Powles , M. S. van der Heijden , A. V. Balar , A. Necchi , et al. 2016 Atezolizumab in patients with locally advanced and metastatic urothelial carcinoma who have progressed following treatment with platinum‐based chemotherapy: a single‐arm, multicentre, phase 2 trial. Lancet 387:1909–1920. 26952546 10.1016/S0140-6736(16)00561-4 PMC5480242

[13] Plimack, E. R. , J. Bellmunt , S. Gupta , R. Berger , L. Q. Chow , J. Juco , et al. 2017 Safety and activity of pembrolizumab in patients with locally advanced or metastatic urothelial cancer (KEYNOTE‐012): a non‐randomised, open‐label, phase 1b study. Lancet Oncol. 18:212–220. 28081914 10.1016/S1470-2045(17)30007-4

[14] Kohler, G. , and C. Milstein . 1976 Derivation of specific antibody‐producing tissue culture and tumor lines by cell fusion. Eur. J. Immunol. 6:511–519. 825377 10.1002/eji.1830060713

[15] Tanaka, M. , J. R. Gee , J. De La Cerda , C. J. Rosser , J. H. Zhou , W. F. Benedict , et al. 2003 Noninvasive detection of bladder cancer in an orthotopic murine model with green fluorescence protein cytology. J. Urol. 170:975–978. 12913753 10.1097/01.ju.0000073209.65128.c1

[16] Cumashi, A. , N. Tinari , C. Rossi , R. Lattanzio , C. Natoli , M. Piantelli , et al. 2008 sunitinib malate (su‐11248) alone or in combination with low‐dose docetaxel inhibits the growth of du‐145 prostate cancer xenografts. Cancer Lett. 270:229–233. 18586384 10.1016/j.canlet.2008.05.007

[17] Livak, K. J. , and T. D. Schmittgen . 2001 analysis of relative gene expression data using real‐time quantitative pcr and the 2(‐delta delta c(t)) method. Methods 25:402–408. 11846609 10.1006/meth.2001.1262

[18] Dalbagni, G. , P. Russo , B. Bochner , L. Ben‐Porat , J. Sheinfeld , P. Sogani , et al. 2006 Phase II trial of intravesical gemcitabine in bacille Calmette‐Guerin‐refractory transitional cell carcinoma of the bladder. J. Clin. Oncol. 24:2729–2734. 16782913 10.1200/JCO.2005.05.2720

[19] Barlow, L. J. , J. M. McKiernan , and M. C. Benson . 2013 Long‐term survival outcomes with intravesical docetaxel for recurrent nonmuscle invasive bladder cancer after previous bacillus Calmette‐Guerin therapy. J. Urol. 189:834–839. 23123371 10.1016/j.juro.2012.10.068

[20] Lightfoot, A. J. , B. N. Breyer , H. M. Rosevear , B. A. Erickson , B. R. Konety , and M. A. O'Donnell . 2014 Multi‐institutional analysis of sequential intravesical gemcitabine and mitomycin C chemotherapy for non‐muscle invasive bladder cancer. Urol. Oncol.. 32:35.e15–39. 10.1016/j.urolonc.2013.01.009 PMC4113080 23510863

[21] Nepple, K. G. , A. J. Lightfoot , H. M. Rosevear , M. A. O'Donnell , and D. L. Lamm . 2010 Bacillus Calmette‐Guerin with or without interferon alpha‐2b and megadose versus recommended daily allowance vitamins during induction and maintenance intravesical treatment of nonmuscle invasive bladder cancer. J. Urol. 184:1915–1919. 20846688 10.1016/j.juro.2010.06.147

[22] Ponta, H. , L. Sherman , and P. A. Herrlich . 2003 CD44: from adhesion molecules to signalling regulators. Nat. Rev. Mol. Cell Biol. 4:33–45. 12511867 10.1038/nrm1004

[23] van Weering, D. H. , P. D. Baas , and J. L. Bos . 1993 A PCR‐based method for the analysis of human CD44 splice products. PCR Methods Appl. 3:100–106. 7505677 10.1101/gr.3.2.100

[24] Sneath, R. J. , and D. C. Mangham . 1998 The normal structure and function of CD44 and its role in neoplasia. Mol. Pathol. 51:191–200. 9893744 10.1136/mp.51.4.191 PMC395635

[25] Naor, D. , S. B. Wallach‐Dayan , M. A. Zahalka , and R. V. Sionov . 2008 Involvement of CD44, a molecule with a thousand faces, in cancer dissemination. Semin. Cancer Biol. 18:260–267. 18467123 10.1016/j.semcancer.2008.03.015

[26] Johnson, P. , A. Maiti , K. L. Brown , and R. Li . 2000 A role for the cell adhesion molecule CD44 and sulfation in leukocyte‐endothelial cell adhesion during an inflammatory response? Biochem. Pharmacol. 59:455–465. 10660111 10.1016/s0006-2952(99)00266-x

[27] Kakehashi, A. , N. Ishii , E. Sugihara , M. Gi , H. Saya , and H. Wanibuchi . 2016 CD44 variant 9 is a potential biomarker of tumor initiating cells predicting survival outcome in hepatitis C virus‐positive patients with resected hepatocellular carcinoma. Cancer Sci. 107:609–618. 26882440 10.1111/cas.12908 PMC4970827

[28] Hirata, K. , H. Suzuki , H. Imaeda , J. Matsuzaki , H. Tsugawa , O. Nagano , et al. 2013 CD44 variant 9 expression in primary early gastric cancer as a predictive marker for recurrence. Br. J. Cancer 109:379–386. 23778530 10.1038/bjc.2013.314 PMC3721391

[29] Dalerba, P. , S. J. Dylla , I. K. Park , R. Liu , X. Wang , R. W. Cho , et al. 2007 Phenotypic characterization of human colorectal cancer stem cells. Proc. Natl Acad. Sci. U S A 104:10158–10163. 17548814 10.1073/pnas.0703478104 PMC1891215

[30] Al‐Hajj, M. , M. S. Wicha , A. Benito‐Hernandez , S. J. Morrison , and M. F. Clarke . 2003 Prospective identification of tumorigenic breast cancer cells. Proc. Natl Acad. Sci. U S A 100:3983–3988. 12629218 10.1073/pnas.0530291100 PMC153034

[31] Kaufmann, M. , K. H. Heider , H. P. Sinn , G. von Minckwitz , H. Ponta , and P. Herrlich . 1995 CD44 variant exon epitopes in primary breast cancer and length of survival. Lancet 345:615–619. 7534855 10.1016/s0140-6736(95)90521-9

[32] Wang, Z. , Q. Wang , Q. Wang , Y. Wang , and J. Chen . 2017 Prognostic significance of CD24 and CD44 in breast cancer: a meta‐analysis. Int. J. Biol. Markers 32:e75–e82. 27470135 10.5301/jbm.5000224

[33] Iczkowski, K. A. , J. H. Shanks , and D. G. Bostwick . 1998 Loss of CD44 variant 6 expression differentiates small cell carcinoma of urinary bladder from urothelial (transitional cell) carcinoma. Histopathology 32:322–327. 9602328 10.1046/j.1365-2559.1998.00398.x

[34] Lipponen, P. , S. Aaltoma , V. M. Kosma , M. Ala‐Opas , and M. Eskelinen . 1998 Expression of CD44 standard and variant‐v6 proteins in transitional cell bladder tumours and their relation to prognosis during a long‐term follow‐up. J. Pathol. 186:157–164. 9924431 10.1002/(SICI)1096-9896(1998100)186:2<157::AID-PATH169>3.0.CO;2-M

[35] Hagiwara, M. , E. Kikuchi , T. Kosaka , S. Mikami , H. Saya , and M. Oya . 2016 Variant isoforms of CD44 expression in upper tract urothelial cancer as a predictive marker for recurrence and mortality. Urol. Oncol. 34:337.e319–326. 10.1016/j.urolonc.2016.03.015 27133224

[36] Kobayashi, K. , H. Matsumoto , H. Matsuyama , N. Fujii , R. Inoue , Y. Yamamoto , et al. 2016 Clinical significance of CD44 variant 9 expression as a prognostic indicator in bladder cancer. Oncol. Rep. 36:2852–2860. 27599396 10.3892/or.2016.5061

[37] Brooks, C. L. , A. Schietinger , S. N. Borisova , P. Kufer , M. Okon , T. Hirama , et al. 2010 Antibody recognition of a unique tumor‐specific glycopeptide antigen. Proc. Natl Acad. Sci. U S A 107:10056–10061. 20479270 10.1073/pnas.0915176107 PMC2890472

[38] Park, J. H. , T. Nishidate , K. Kijima , T. Ohashi , K. Takegawa , T. Fujikane , et al. 2010 Critical roles of mucin 1 glycosylation by transactivated polypeptide N‐acetylgalactosaminyltransferase 6 in mammary carcinogenesis. Cancer Res. 70:2759–2769. 20215525 10.1158/0008-5472.CAN-09-3911

[39] Mori, H. , T. Tomari , N. Koshikawa , M. Kajita , Y. Itoh , H. Sato , et al. 2002 CD44 directs membrane‐type 1 matrix metalloproteinase to lamellipodia by associating with its hemopexin‐like domain. EMBO J. 21:3949–3959. 12145196 10.1093/emboj/cdf411 PMC126155

[40] Hanley, W. D. , S. L. Napier , M. M. Burdick , R. L. Schnaar , R. Sackstein , and K. Konstantopoulos . 2006 Variant isoforms of CD44 are P‐ and L‐selectin ligands on colon carcinoma cells. Faseb J. 20:337–339. 16352650 10.1096/fj.05-4574fje

[41] Alves, C. S. , S. Yakovlev , L. Medved , and K. Konstantopoulos . 2009 Biomolecular characterization of CD44‐fibrin(ogen) binding: distinct molecular requirements mediate binding of standard and variant isoforms of CD44 to immobilized fibrin(ogen). J. Biol. Chem. 284:1177–1189. 19004834 10.1074/jbc.M805144200 PMC2613610

[42] Tang, L. , J. Yang , X. Tang , W. Ying , X. Qian , and F. He . 2010 The DC‐SIGN family member LSECtin is a novel ligand of CD44 on activated T cells. Eur. J. Immunol. 40:1185–1191. 20127679 10.1002/eji.200939936

[43] Orian‐Rousseau, V. , L. Chen , J. P. Sleeman , P. Herrlich , and H. Ponta . 2002 CD44 is required for two consecutive steps in HGF/c‐Met signaling. Genes Dev. 16:3074–3086. 12464636 10.1101/gad.242602 PMC187488

[44] Song, G. , X. Liao , L. Zhou , L. Wu , Y. Feng , and Z. C. Han . 2004 HI44a, an anti‐CD44 monoclonal antibody, induces differentiation and apoptosis of human acute myeloid leukemia cells. Leuk. Res. 28:1089–1096. 15289023 10.1016/j.leukres.2004.02.005

[45] Jin, L. , K. J. Hope , Q. Zhai , F. Smadja‐Joffe , and J. E. Dick . 2006 Targeting of CD44 eradicates human acute myeloid leukemic stem cells. Nat. Med. 12:1167–1174. 16998484 10.1038/nm1483

[46] Yasuda, M. , Y. Tanaka , K. Fujii , and K. Yasumoto . 2001 CD44 stimulation down‐regulates Fas expression and Fas‐mediated apoptosis of lung cancer cells. Int. Immunol. 13:1309–1319. 11581176 10.1093/intimm/13.10.1309

[47] Naor, D. , S. Nedvetzki , I. Golan , L. Melnik , and Y. Faitelson . 2002 CD44 in cancer. Crit. Rev. Clin. Lab. Sci. 39:527–579. 12484499 10.1080/10408360290795574

[48] Martin, T. A. , G. Harrison , R. E. Mansel , and W. G. Jiang . 2003 The role of the CD44/ezrin complex in cancer metastasis. Crit. Rev. Oncol./Hematol. 46:165–186. 10.1016/s1040-8428(02)00172-5 12711360

[49] Li, C. , Y. Du , Z. Yang , L. He , Y. Wang , L. Hao , et al. 2016 GALNT1‐mediated glycosylation and activation of sonic Hedgehog signaling maintains the self‐renewal and tumor‐initiating capacity of bladder cancer stem cells. Cancer Res. 76:1273–1283. 26676748 10.1158/0008-5472.CAN-15-2309

[50] Li, C. , Z. Yang , Y. Du , H. Tang , J. Chen , D. Hu , et al. 2014 BCMab1, a monoclonal antibody against aberrantly glycosylated integrin alpha3beta1, has potent antitumor activity of bladder cancer in vivo. Clin. Cancer Res. 20:4001–4013. 25002124 10.1158/1078-0432.CCR-13-3397

[51] Takada, Y. , X. Ye , and S. Simon . 2007 The integrins. Genome Biol. 8:215. 17543136 10.1186/gb-2007-8-5-215 PMC1929136

[52] Zhao, Q. , H. Wang , M. Yang , D. Yang , Y. Zuo , and J. Wang . 2013 Expression of a tumor‐associated gene, LASS2, in the human bladder carcinoma cell lines BIU‐87, T24, EJ and EJ‐M3. Exp. Ther. Med. 5:942–946. 23407876 10.3892/etm.2013.892 PMC3570257

